# Supplementary material for: Cluster and survival analysis of UK biobank data reveals associations between physical multimorbidity clusters and subsequent depression
Source: Commun Med (Lond). 2025 May 13;5:156. doi: 10.1038/s43856-025-00825-7 (PMC12075648; doi:10.1038/s43856-025-00825-7)
Supplement: Supplementary file 2 — Supplementary Information [file 43856_2025_825_MOESM2_ESM.pdf]

Supplementary Information

Supplementary Figure 1. Flow diagram explaining data filtration steps.

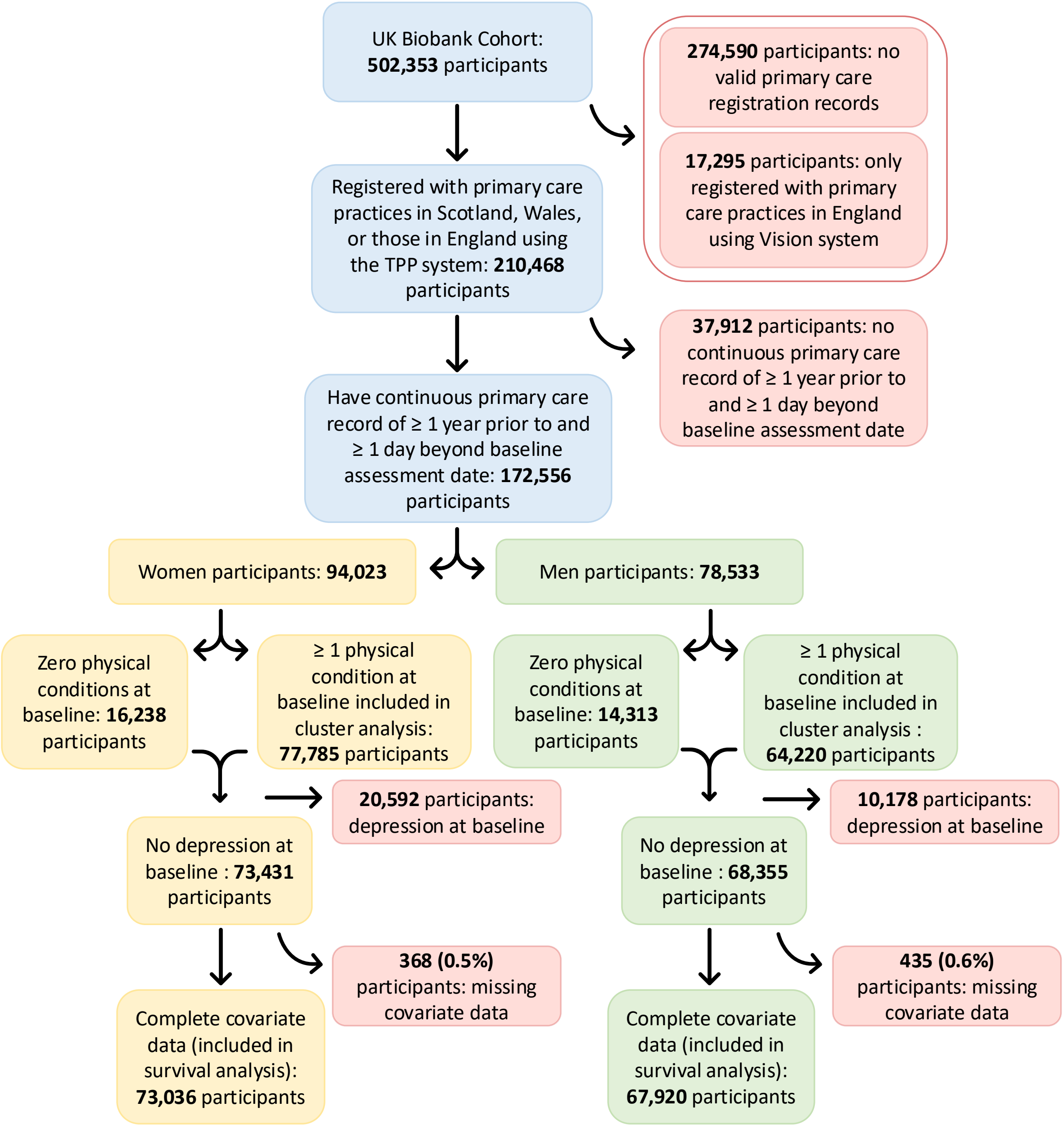

Excluding participants who withdrew permission for their data to be included in research before 13 October 2023.

Supplementary Note 1. Methodological Descriptions.

To identify a clustering method best suited for our data, we explored several combinations of metrics and methods which were, in theory, capable of handling the binary nature of the morbidity data. Specifically, we used the following metrics within this study:

- **Hamming distance** [1]: This is a dissimilarity metric denoting the number of mismatching categories between two objects. It is formally defined as [2]:
$$d_{hamming}(X,Y) = \sum_{j=1}^{m} \delta(x_j, y_j) \,,$$
where:
$$\delta(x_j, y_j) = \begin{cases} 0 & if\ x_j = y_j \\ 1 & if\ x_j \neq y_j \end{cases}.$$
- **cosine similarity** [3]: This is a measure which was originally used to indicate how similar two vector angles are to one another. For binary data, it can be understood as the number of true, or positive features are shared between two objects, divided by the product of the number of true objects from each object. Formally, it is defined as [4]:
$$s_{cosine}(X,Y) = \frac{X \cdot Y}{\|X\| \cdot \|Y\|}$$

With these metrics, we explored the following four clustering methods. Each of these four methods has different properties. For instance, *k*-modes assigns groups based on similarity between the participants of a cluster [5,6], whereas *k*-medoids assigns groups based on similarity to a *single, representative* participant of each cluster [7]. LCA is unique because it gives the *probabilities* of each participant belonging to each cluster [8]. These three methods, though, require prior specification of cluster number, which is typically unknown. In contrast, AHC does not require any pre-specified cluster number; instead, cluster number is determined empirically. However, AHC is limited in that it assumes the data can be separated hierarchically [9].

We formally define each of the four clustering approaches below:

- ***k*-modes** [5,6]: This uses the same methodology as *k*-means clustering [10], but the centroid of each cluster is defined on the number of matching categories between data points, computed via the Hamming distance [1]. In other words, the centroid represents the mode of the cluster, rather than the mean [5,6]. Centroid initialization was performed via the frequency-based *Huang* metric [5].
- ***k*-medoids** [7]**:** As above, this uses the same methodology as *k*-means clustering [10], but the centroid is an actual data point acting as the "median", computed via cosine similarity.
- **Latent Class Analysis (LCA)** [8]**:** This aims to find groups or subtypes of cases (latent classes) in multivariate categorical data. It gives probabilities of class membership, rather than concrete class assignments, which are unique, so the user can see the likelihood that a data point truly belongs to its assigned class [8].
- **agglomerative hierarchical clustering (AHC)** [9]**:** This is best understood as a "bottom-up" approach in which samples start out alone, then merge to form larger and larger clusters [11]. We used a *complete* linkage (the maximum distance between points in two clusters [12]), computed via Hamming distance [1].

Finally, we assessed cluster performance, including separation and overlap, with the following three performance metrics:

- **Calinski and Harabasz score** [13]: This is the ratio of between-cluster dispersion to within-cluster dispersion. A higher Calinski and Harabasz score indicates better performance.
- **Davies Bouldin score** [14]: This is a measure of cluster similarity to each cluster's most similar cluster. A Davies Bouldin score closer to zero indicates better performance.
- **Silhouette score** [15]: This is a measure of cluster fit which accounts for the mean distance between points in each individual cluster as well as the mean distance to points in the closest neighboring cluster. A silhouette score closer to one indicates better performance. Hamming distance was utilized as the distance metric.

The best similarity or dissimilarity metrics were selected for *k*-modes, *k*-medoids, and AHC by testing each of them upon a random selection of participants (1,417). The *k*-modes method used with an alternative initialization technique, called the *Cao* metric [6], resulted in some clusters containing less than ten participants, while others contained hundreds. Such imbalance is uninformative for our purposes. Similarly, *k*-medoids with Hamming distance and *Jaccard similarity* [16], another similarity metric, resulted in several empty clusters. Finally, AHC with other metrics and linkage types resulted in poor separation between clusters, with high overlap between branches. These issues were not present with the specified metrics.

Supplementary Note 2. Bubble Heatmap.

The *bubble heatmap* places ARF values on a grid in which the *y*-axis contains conditions, the *x*-axis contains clusters, and data points are colored blue (under-representation) or red (over-representation) at each intersection. The magnitude of under- or over-representation is indicated by the size of the data point, or *bubble.* Points are not statistically significant, as determined by the Fisher's Exact test[17], were omitted. Therefore, conditions with no significant values are omitted entirely from the *y*-axis.

Notably, for visualisation purposes, the ARF values are adjusted so that values denoting under-representation (between zero and one) were mapped to a similar scale as those denoting over-representation (values greater than one). Specifically, we used the following function, in which *x* denotes the original ARF value:

$$f(x) = \frac{2\,(x - 1)}{(x + 1)}$$

Supplementary Table 1. Cluster labels for the *whole* cohort.

**Cluster Label**	**Num. conditions significantly over-represented**	**Num. conditions significantly under-represented**	**Explanation**
Very extensive morbidity	41	4	41 conditions are significantly over-represented with no stand-out conditions or groups.
Migraine	7	21	Migraine has large over-representation with relatively small over-representation of six other conditions.
Respiratory	8	19	Four respiratory conditions have large over-representation, with four other conditions significantly over-represented.
Respiratory w/o other	3	29	Three respiratory conditions are significantly over-represented, with widespread significant under-representation of other conditions.
CVD + diabetes	16	27	Nine cardiovascular disease (CVD) conditions and three diabetes types are significantly over-represented but, unlike 'very extensive morbidity', many other conditions are under-represented.
Mixed including cancer	23	21	Twenty-three conditions, including cancers, are significantly over-represented with no stand-out conditions or groups, but CVD and respiratory conditions are significantly under-represented. However, over-represented conditions are relatively rare so this group is relatively healthy.
Healthy + rhinitis	1	41	Allergic and chronic rhinitis is significantly over-represented, with significant under-representation of 41 other conditions
Macular degeneration + diabetes	5	6	Diabetes and eye conditions significantly over-represented, of which macular degeneration has the highest prevalence.

Supplementary Table 2. Cluster labels for the *women-only* cohort.

**Cluster Label**	**No. of conditions significantly over-represented**	**No. of conditions significantly under-represented**	**Explanation**
Very extensive morbidity	34	0	34 conditions are significantly over-represented with no stand-out conditions or groups.
Extensive morbidity	23	2	23 conditions are significantly over-represented with no stand-out conditions or groups and two relatively small under-representation of two conditions.
Respiratory	4	14	Four respiratory conditions have large over-representation, with no other significantly over-represented conditions.
Digestive	5	3	Four digestive conditions are significantly over-represented, two of which are largely over-represented. Additionally, one CVD condition is over-represented, but the other 11 CVD conditions are not.
MSK	7	11	Five musculoskeletal (MSK) conditions are significantly over-represented, with relatively small over-representation of two other conditions and under-representation of 11 other conditions.
CVD + diabetes	15	18	Nine CVD conditions and three diabetes types are significantly over-represented, but, unlike ‘very extensive morbidity’, many other conditions are under-represented.
Mixed including cancer	17	18	Seventeen conditions, including cancers, are significantly over-represented with no stand-out conditions or groups, but CVD, respiratory and MSK are significantly under-represented. However, over-represented conditions are relatively rare, so this group is relatively healthy.
Healthy + rhinitis	1	44	Allergic and chronic rhinitis is significantly over-represented, with significant under-representation of 44 other conditions.

Supplementary Table 3. Cluster labels for the *men-only* cohort.

Cluster Label	No. of conditions significantly over-represented	No. of conditions significantly under-represented	Explanation
Very extensive morbidity	29	3	29 conditions are significantly over-represented with no stand-out conditions or groups. While three conditions have relatively small under-representation, there are no stand-out conditions or groups amongst them either.
MSK + others	14	7	Four musculoskeletal conditions are significantly over represented, but additionally 10 other conditions with no stand out pattern (unlike the Women-only *MSK* cluster, which is more purely MSK).
Respiratory + gout + male genitourinary	6	1	Three respiratory conditions, gout, and two genitourinary conditions affecting primarily men have large over-representation, while only one condition has relatively small under-representation.
Digestive	6	6	Four digestive conditions have large over-representation, with two other conditions significantly over-represented.
CVD + diabetes	11	20	Eight CVD conditions and two diabetes types are significantly over-represented, in addition to one other condition, but, unlike 'very extensive morbidity', many other conditions are under-represented.
CVD + CKD + gout	11	5	Seven CVD conditions are significantly over-represented. Additionally, gout and chronic renal/kidney disease (CKD) have large over-representation.
Mixed including cancer	19	18	Nineteen conditions, including cancers, are significantly over-represented with no stand-out conditions or groups, but CVD, respiratory and MSK are significantly under-represented. However, the over-represented conditions are relatively rare, so this group is relatively healthy.
Healthy + rhinitis	2	42	Allergic and chronic rhinitis as well as Asthma are significantly over-represented, with significant under-representation of 42 other conditions.

Supplementary Figure 2. Prevalence values per cluster and condition.

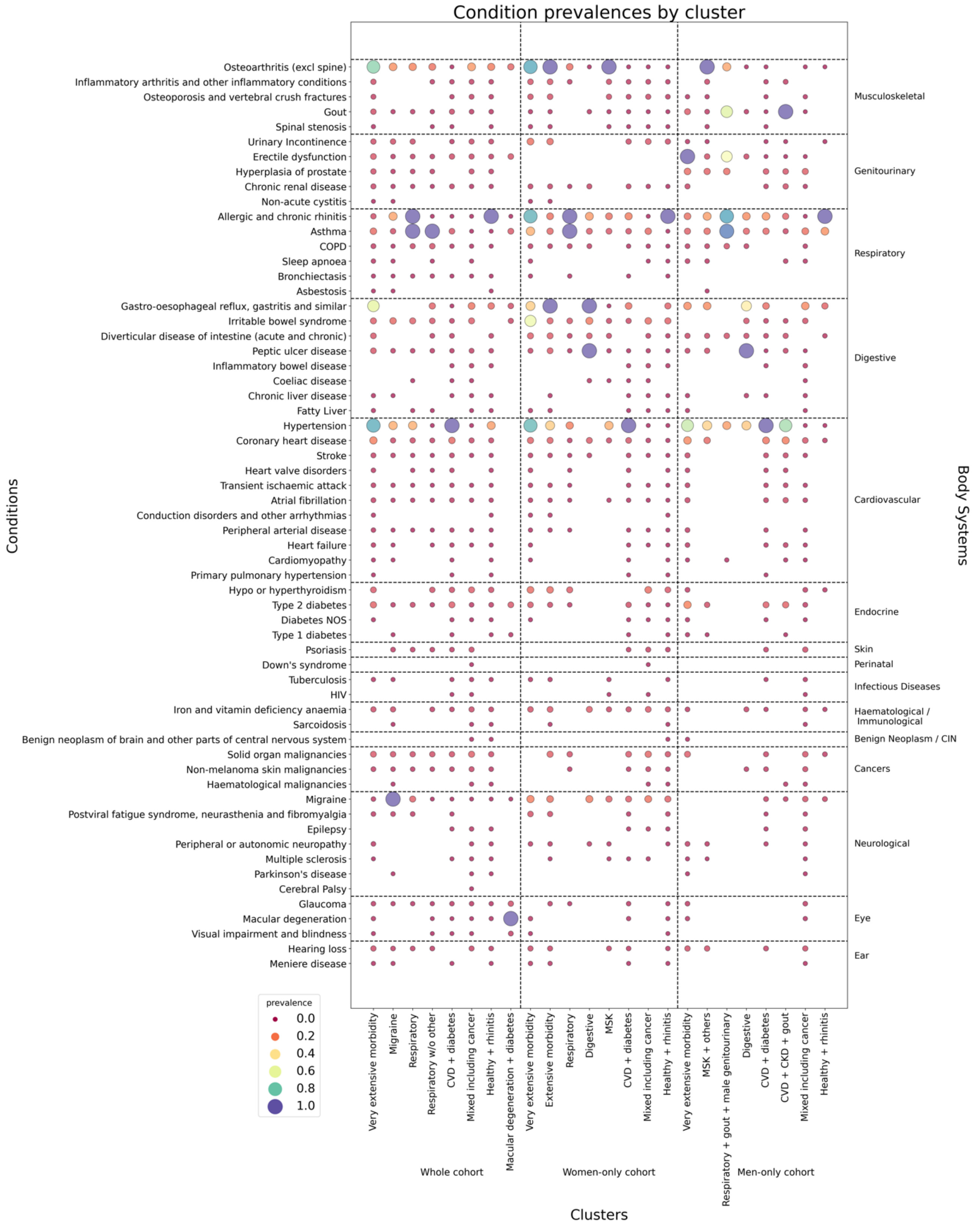

Bubble sizes and hues represent the prevalence of each condition within each respective cluster (n=142,005 participants in the whole cohort, 77,785 participants in the women-only cohort 64,220 participants in the men-only cohort). Hues closer to red indicate low prevalence, whereas hues closer to purple indicate high prevalence. Conditions with a prevalence of zero are omitted.

Supplementary Figure 3. Time-to-depression diagnosis for each cluster in each of the *k*-modes models.

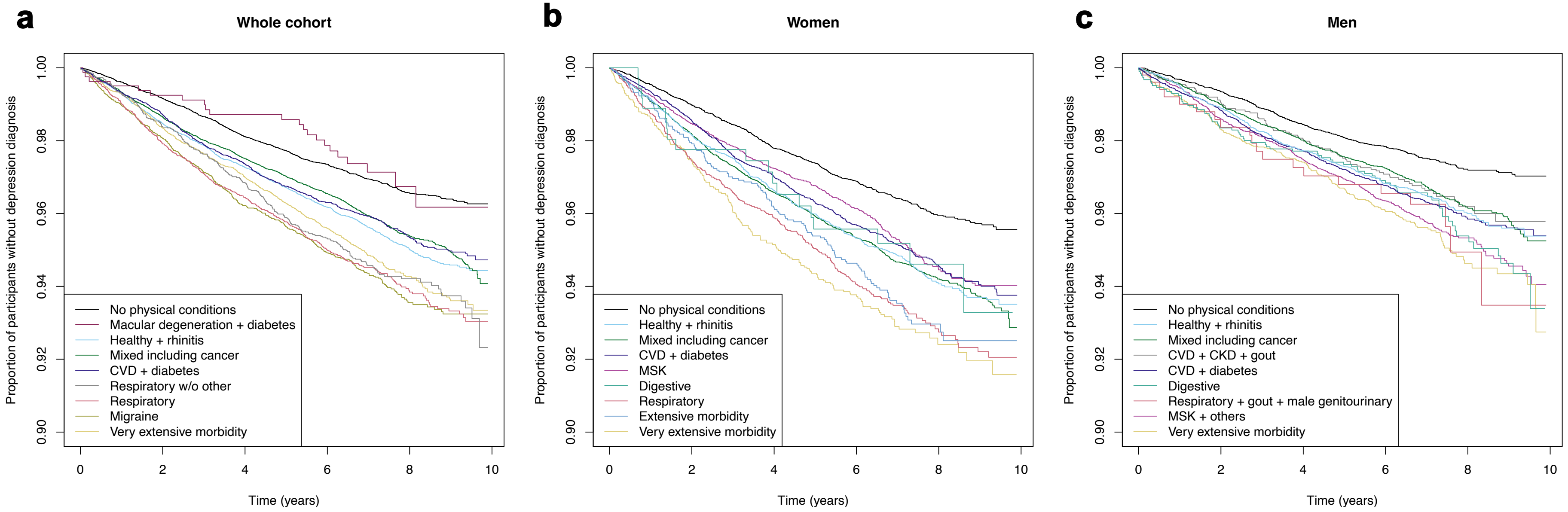

a Time-to-depression diagnosis for each cluster in the k-modes model for the whole cohort. **b** Time-to-depression diagnosis for each cluster in the k-modes model for the women-only cohort. **c** Time-to-depression diagnosis for each cluster in the k-modes model for the men-only cohort. All *y*-axes are cropped for better resolution between curves.

Supplementary References

1. Hamming, R. W. Error Detecting and Error Correcting Codes. *Bell System Technical Journal* 29, 147–160 (1950).
2. Kaufman, L. and P. J. R. *Finding Groups in Data: An Introduction to Cluster Analysis*. (John Wiley & Sons, 2009).
3. Salton, G., Wong, A. & Yang, C. S. A vector space model for automatic indexing. *Commun ACM* 18, 613–620 (1975).
4. Manning, C. D., Raghavan, P. & Schütze, H. *Introduction to Information Retrieval*. (Cambridge University Press, 2008).
5. Huang, Z. Clustering large data sets with mixed numeric and categorical values. in *Proceedings of the 1st pacific-asia conference on knowledge discovery and data mining,(PAKDD)* 21–34 (1997).
6. Cao, F., Liang, J. & Bai, L. A new initialization method for categorical data clustering. *Expert Syst Appl* 36, 10223–10228 (2009).
7. Jin Xin and Han, J. K-Medoids Clustering. *Encyclopedia of Machine Learning* 564–565 (2010) doi:10.1007/978-0-387-30164-8_426.
8. Weller, B. E., Bowen, N. K. & Faubert, S. J. Latent class analysis: a guide to best practice. *Journal of Black Psychology* 46, 287–311 (2020).
9. Sasirekha, K. & Baby, P. Agglomerative hierarchical clustering algorithm-a. *International Journal of Scientific and Research Publications* 83, 83 (2013).
10. MacQueen, J. Classification and analysis of multivariate observations. in *5th Berkeley Symp. Math. Statist. Probability* 281–297 (1967).
11. Robert, L. Thorndike."Who Belongs in the Family?". *Psychometrika* 18, 267–276 (1953).
12. Nielsen, F. Hierarchical Clustering. in 195–211 (2016). doi:10.1007/978-3-319-21903-5_8.
13. Caliński, T. & Harabasz, J. A dendrite method for cluster analysis. *Communications in Statistics-theory and Methods* 3, 1–27 (1974).
14. Davies, D. L. & Bouldin, D. W. A cluster separation measure. *IEEE Trans Pattern Anal Mach Intell* 2, 224–227 (1979).
15. Rousseeuw, P. J. Silhouettes: A graphical aid to the interpretation and validation of cluster analysis. *J Comput Appl Math* 20, 53–65 (1987).
16. Jaccard, P. Étude comparative de la distribution florale dans une portion des Alpes et des Jura. *Bull Soc Vaudoise Sci Nat* 37, 547–579 (1901).
17. Guide, P. Fisher's Exact Test. Preprint at https://www.pathwaycommons.org/guide/primers/statistics/fishers_exact_test/.
