# Supplementary material for: Cluster and survival analysis of UK biobank data reveals associations between physical multimorbidity clusters and subsequent depression
Source: Commun Med (Lond). 2025 May 13;5:156. doi: 10.1038/s43856-025-00825-7 (PMC12075648; doi:10.1038/s43856-025-00825-7)
Supplement: Supplementary file 3 — Description of Additional Supplementary File [file 43856_2025_825_MOESM3_ESM.pdf]

Description Of Additional Supplementary File

File name: Supplementary Data 1
Description: 69 physical conditions with corresponding body systems.

File name: Supplementary Data 2.
Description: Excel sheets containing ARF values and adjusted p-values per condition and cluster. Statistically significant values ($p < 0.05$) are denoted in bold.

File name: Supplementary Data 3
Description: Excel file containing boxplot metadata (Quartiles and interquartile range (IQR) values) corresponding to Figure 2, panel d (whole cohort clusters).

File name: Supplementary Data 4
Description: Excel file containing boxplot metadata (Quartiles and interquartile range (IQR) values) corresponding to Figure 2, panel e (women-only cohort clusters).

File name: Supplementary Data 5
Description: Excel file containing boxplot metadata (Quartiles and interquartile range (IQR) values) corresponding to Figure 2, panel f (men-only cohort clusters).
