# Supplementary material for: Cluster and survival analysis of UK biobank data reveals associations between physical multimorbidity clusters and subsequent depression
Source: Commun Med (Lond). 2025 May 13;5:156. doi: 10.1038/s43856-025-00825-7 (PMC12075648; doi:10.1038/s43856-025-00825-7)
Supplement: Supplementary file 4 — Supplementary Data 1 [file 43856_2025_825_MOESM4_ESM.docx]

| **System** | **Condition** |
| --- | --- |
| Skin conditions | Psoriasis |
| Perinatal conditions | Down's syndrome |
| Neurological conditions | Migraine |
|  | Peripheral or autonomic neuropathy |
|  | Epilepsy |
|  | Cerebral Palsy |
|  | Motor neuron disease |
|  | Multiple sclerosis |
|  | Myasthenia gravis |
|  | Parkinson's disease |
|  | Postviral fatigue syndrome, neurasthenia and fibromyalgia |
| Musculoskeletal conditions | Inflammatory arthritis and other inflammatory conditions |
|  | Gout |
|  | Osteoporosis and vertebral crush fractures |
|  | Osteoarthritis (excl spine) |
|  | Spinal stenosis |
| Mental Health Disorders | Dementia |
| Infectious Diseases  Infectious Diseases | Tuberculosis |
|  | HIV |
| Haematological/Immunological conditions | Sarcoidosis |
|  | Iron and vitamin deficiency anaemia |
|  | Immunodeficiencies |
|  | Sickle-cell anaemia |
|  | Thalassaemia |
| Diseases of the Respiratory System | Sleep apnoea |
|  | COPD |
|  | Bronchiectasis |
|  | Asthma |
|  | Asbestosis |
|  | Allergic and chronic rhinitis |
| Diseases of the Genitourinary system | Chronic renal disease |
|  | Urinary Incontinence |
|  | Erectile dysfunction |
|  | Non-acute cystitis |
|  | Hyperplasia of prostate |
| Diseases of the Eye | Visual impairment and blindness |
|  | Macular degeneration |
|  | Glaucoma |
| Diseases of the Endocrine System | Hypo or hyperthyroidism |
|  | Addisons disease |
|  | Cystic Fibrosis |
|  | Diabetes NOS |
|  | Type 1 diabetes |
|  | Type 2 diabetes |
| Diseases of the Ear | Meniere disease |
|  | Hearing loss |
| Diseases of the Digestive System | Fatty Liver |
|  | Chronic liver disease |
|  | Peptic ulcer disease |
|  | Irritable bowel syndrome |
|  | Diverticular disease of intestine (acute and chronic) |
|  | Gastro-oesophageal reflux, gastritis and similar |
|  | Coeliac disease |
|  | Inflammatory bowel disease |
| Diseases of the Circulatory System | Conduction disorders and other arrhythmias |
|  | Coronary heart disease |
|  | Cardiomyopathy |
|  | Heart valve disorders |
|  | Atrial fibrillation |
|  | Transient ischaemic attack |
|  | Peripheral arterial disease |
|  | Hypertension |
|  | Heart failure |
|  | Primary pulmonary hypertension |
|  | Stroke |
| Cancers | Solid organ malignancies |
|  | Haematological malignancies |
|  | Non-melanoma skin malignancies |
| Benign Neoplasm/CIN | Benign neoplasm of brain and other parts of central nervous system |
