# Supplementary material for: Cluster and survival analysis of UK biobank data reveals associations between physical multimorbidity clusters and subsequent depression
Source: Commun Med (Lond). 2025 May 13;5:156. doi: 10.1038/s43856-025-00825-7 (PMC12075648; doi:10.1038/s43856-025-00825-7)
Supplement: Supplementary file 9 — REPORTING SUMMARY [file 43856_2025_825_MOESM9_ESM.pdf]

nature portfolio

Corresponding author(s): Lauren Nicole DeLong

Last updated by author(s): 17/02/2025

Reporting Summary

Nature Portfolio wishes to improve the reproducibility of the work that we publish. This form provides structure for consistency and transparency in reporting. For further information on Nature Portfolio policies, see our Editorial Policies and the Editorial Policy Checklist.

Statistics

For all statistical analyses, confirm that the following items are present in the figure legend, table legend, main text, or Methods section.

n/a	Confirmed	
☐	☒	The exact sample size (*n*) for each experimental group/condition, given as a discrete number and unit of measurement
☒	☐	A statement on whether measurements were taken from distinct samples or whether the same sample was measured repeatedly
☐	☒	The statistical test(s) used AND whether they are one- or two-sided<br>*Only common tests should be described solely by name; describe more complex techniques in the Methods section.*
☐	☒	A description of all covariates tested
☐	☒	A description of any assumptions or corrections, such as tests of normality and adjustment for multiple comparisons
☐	☒	A full description of the statistical parameters including central tendency (e.g. means) or other basic estimates (e.g. regression coefficient) AND variation (e.g. standard deviation) or associated estimates of uncertainty (e.g. confidence intervals)
☐	☒	For null hypothesis testing, the test statistic (e.g. *F*, *t*, *r*) with confidence intervals, effect sizes, degrees of freedom and *P* value noted<br>*Give P values as exact values whenever suitable.*
☒	☐	For Bayesian analysis, information on the choice of priors and Markov chain Monte Carlo settings
☒	☐	For hierarchical and complex designs, identification of the appropriate level for tests and full reporting of outcomes
☒	☐	Estimates of effect sizes (e.g. Cohen's *d*, Pearson's *r*), indicating how they were calculated

Our web collection on statistics for biologists contains articles on many of the points above.

Software and code

Policy information about availability of computer code

Data collection	No code was used to collect the data. Data was downloaded from the UK Biobank following a successful application for access.
Data analysis	All analyses except the survival analysis was conducted through software made publicly available at: https://github.com/laurendelong21/clusterMed . The Cox proportional-hazards survival analyses were made through the R package called survival.

For manuscripts utilizing custom algorithms or software that are central to the research but not yet described in published literature, software must be made available to editors and reviewers. We strongly encourage code deposition in a community repository (e.g. GitHub). See the Nature Portfolio guidelines for submitting code & software for further information.

Data

Policy information about availability of data

All manuscripts must include a data availability statement. This statement should provide the following information, where applicable:

- Accession codes, unique identifiers, or web links for publicly available datasets
- A description of any restrictions on data availability
- For clinical datasets or third party data, please ensure that the statement adheres to our policy

The UK Biobank data is not openly available to protect the privacy of participants. Researchers can register for access here: https://www.ukbiobank.ac.uk/enable-your-research/register . The numerical values underlying main text Figure 1, including adjusted p-values, are included in Supplementary Data 2. The numerical values underlying Figure 2, panels d-f, are included in Supplementary Data 3-5. All other data underlying Figures 2-3 are included in Tables 1-2.

Human research participants

Policy information about studies involving human research participants and Sex and Gender in Research.

Reporting on sex and gender	In our study, we stratify participants by sex (women and men). Sex in the UK Biobank is determined by both NHS records and the participants' self-reports upon enrollment. Participants provided written informed consent for linkage to national datasets. We applied all described methods to each of three cohorts: one containing participants from both men and women, one with women participants only, and one with men participants only. We did so to investigate any sex-specific differences, which we comment on in the manuscript.
Population characteristics	Participants were aged 37-73. 54% of the included participants were women and 46% were men. A large majority of participants were Caucasian/white.
Recruitment	We did not perform recruiting; data came from the UK Biobank. Notably, data are collected from volunteers who are middle-aged and generally more affluent than the UK average. Additionally, people from ethnic minorities are somewhat under-represented. These factors could limit the generalizability of our findings to the British and worldwide populations.
Ethics oversight	The UK Biobank has ethical approval from the NHS North West Research Ethics Committee (reference: 21/NW/0157).

Note that full information on the approval of the study protocol must also be provided in the manuscript.

Field-specific reporting

Please select the one below that is the best fit for your research. If you are not sure, read the appropriate sections before making your selection.

☒ Life sciences ☐ Behavioural & social sciences ☐ Ecological, evolutionary & environmental sciences

For a reference copy of the document with all sections, see nature.com/documents/nr-reporting-summary-flat.pdf

Life sciences study design

All studies must disclose on these points even when the disclosure is negative.

Sample size	Of 502,353 participants in the UK Biobank, our study population included 172,556 participants with linked data from the GP electronic health record and a continuous primary care record from at least a year before to at least one day beyond their baseline assessment. There were 140,956 participants (73,036 women and 67,920 men) with at least one physical condition at baseline who were included in the clustering analysis. Survival analysis of time to incident depression diagnosis included 141,001 participants (73,036 women and 67,920 men), excluding 30,770 participants with a history of depression at baseline (20,592 women and 10,178 men). In addition to participants included in the clustering analysis, this analysis also included 30,551 participants with no physical conditions at baseline (16,238 women and 14,313 men).
Data exclusions	We excluded participants who had withdrawn consent, and those with INPS Vision GP records from England because the extraction process excluded participants who died prior to data extraction. We also excluded participants who withdrew from the study. Within the clustering analyses, we excluded 16,238 women and 14,313 men who had no physical conditions at baseline. From the survival analyses, we excluded those who already had a depression diagnosis at baseline (20,592 women and 10,178 men) and those who were missing covariate data (368 women and 435 men) .
Replication	Any clustering approaches which are sensitive to variation during initialization were performed five times, independently. Accordingly, the corresponding averages and standard deviations are reported. Approaches which are insensitive to initialization (i.e., perform consistently every time due to the designs of their algorithms) are not repeated.
Randomization	Before clustering, the participants within each cohort were shuffled at random (within the cohort). This ensured that any clustering approaches which could be sensitive to data order were not affected.
Blinding	Blinding was not relevant to this study. The aforementioned randomization process is automated and therefore unaffected by user bias.

Reporting for specific materials, systems and methods

We require information from authors about some types of materials, experimental systems and methods used in many studies. Here, indicate whether each material, system or method listed is relevant to your study. If you are not sure if a list item applies to your research, read the appropriate section before selecting a response.

Materials & experimental systems

n/a	Involved in the study
☒	☐ Antibodies
☒	☐ Eukaryotic cell lines
☒	☐ Palaeontology and archaeology
☒	☐ Animals and other organisms
☒	☐ Clinical data
☒	☐ Dual use research of concern

Methods

n/a	Involved in the study
☒	☐ ChIP-seq
☒	☐ Flow cytometry
☒	☐ MRI-based neuroimaging
